# Single strand conformation polymorphism based SNP and Indel markers for genetic mapping and synteny analysis of common bean (*Phaseolus vulgaris *L.)

**DOI:** 10.1186/1471-2164-10-629

**Published:** 2009-12-23

**Authors:** Carlos H Galeano, Andrea C Fernández, Marcela Gómez, Matthew W Blair

**Affiliations:** 1 Centro Internacional de Agricultura Tropical (CIAT), Apartado Aéreo 6713, Cali, Colombia; 2 Current address: Laboratorio Nacional Interinstitucional de Detección y Monitoreo de Organismos Genéticamente Modificados, Instituto Colombiano Agropecuario. Km 14 Recta Tibaitatá-Mosquera, Colombia

## Abstract

**Background:**

Expressed sequence tags (ESTs) are an important source of gene-based markers such as those based on insertion-deletions (Indels) or single-nucleotide polymorphisms (SNPs). Several gel based methods have been reported for the detection of sequence variants, however they have not been widely exploited in common bean, an important legume crop of the developing world. The objectives of this project were to develop and map EST based markers using analysis of single strand conformation polymorphisms (SSCPs), to create a transcript map for common bean and to compare synteny of the common bean map with sequenced chromosomes of other legumes.

**Results:**

A set of 418 EST based amplicons were evaluated for parental polymorphisms using the SSCP technique and 26% of these presented a clear conformational or size polymorphism between Andean and Mesoamerican genotypes. The amplicon based markers were then used for genetic mapping with segregation analysis performed in the DOR364 × G19833 recombinant inbred line (RIL) population. A total of 118 new marker loci were placed into an integrated molecular map for common bean consisting of 288 markers. Of these, 218 were used for synteny analysis and 186 presented homology with segments of the soybean genome with an e-value lower than 7 × 10^-12^. The synteny analysis with soybean showed a mosaic pattern of syntenic blocks with most segments of any one common bean linkage group associated with two soybean chromosomes. The analysis with *Medicago truncatula *and *Lotus japonicus *presented fewer syntenic regions consistent with the more distant phylogenetic relationship between the galegoid and phaseoloid legumes.

**Conclusion:**

The SSCP technique is a useful and inexpensive alternative to other SNP or Indel detection techniques for saturating the common bean genetic map with functional markers that may be useful in marker assisted selection. In addition, the genetic markers based on ESTs allowed the construction of a transcript map and given their high conservation between species allowed synteny comparisons to be made to sequenced genomes. This synteny analysis may support positional cloning of target genes in common bean through the use of genomic information from these other legumes.

## Background

Single nucleotide polymorphisms (SNPs) and insertion/deletion events (Indels) represent the most frequent polymorphisms found in eukaryotic genomes. For example, in humans the frequency of SNP polymorphisms is one per kilobase and given the large size of the human genome the total number of SNPs has been estimated to be over of 3.1 million [1,2]. Similarly, high SNP frequencies have been reported in plant genomes, especially in out-crossing species, but the discovery process has been slower despite the small genomes of some species. Examples include grapevine (*Vitis vinifera *L.), an out-crossing species, where one SNP occurs every 78 bp [3] or maize (*Zea mays *L.) where the average frequency of SNPs was one every 43 bases in 1,088 maize gene sequences and where Indels were also common [4]. In a self pollinated species such as soybean (*Glycine max *(L.) Merr), the SNP frequency was reported as one SNP every 191 bp in non-coding regions and one SNP every two kilobases in coding regions based on 15 genotypes and 35 genomic or gene fragments [5]. Rice (*Oryza sativa *L.), another inbreeding species, had one SNP every 300 bp in coding regions and one SNP every 37 bp in transposable elements when comparing *indica *and *japonica *subspecies [6] and recently 159,478 high-quality, non-redundant SNPs were found across the entire rice genome [7]. In this study, our interest was to develop SNP and Indel based markers for common bean (*Phaseolus vulgaris *L.), an important legume in terms of food security but one that has been less well studied as it is found mostly in developing countries.

Expressed sequence tag (EST) libraries offer important information for species that have not been sequenced and are a central source of gene-based markers and SNP or Indel polymorphisms. Discovery of these polymorphisms usually involves alignment of sequences obtained from the sequencing of EST libraries from different genotypes of the same species [8] or from re-sequencing of PCR fragments [9]. EST-based markers are valuable because they represent sequences that are transcribed and therefore can potentially be associated with phenotypic differences. Furthermore, EST based markers are often highly conserved between species allowing the construction of transcript maps and synteny comparisons between genomes.

EST analysis in common bean shows that SNP frequency appears to be similar or higher than in other self-pollinating species although fairly few studies have analyzed their relative abundance across different regions of the genome or across the wide diversity of common bean accessions. In a pioneering study for the crop, Ramirez et al. [10] found that SNP frequency in EST sequences from two genotypes of common bean (the Andean G19833 versus the Mesoamerican Negro Jamapa) was 529 SNPs in 214 kb of SNP-containing contigs, with a frequency of one SNP every 387 bp in this inter-genepool comparison. Recently, Gaitán-Solís et al. [11] reported 239 SNPs and 133 Indels in 45 gene-coding and non-coding fragments analyzed in 10 cultivated and wild bean genotypes belonging to the Mesoamerican and Andean gene pools finding an average frequency of one SNP every 88 bp and one Indel every 157 bp. The high frequency of SNPs and overall genotype diversity in common bean makes this species amenable to SNP marker development.

EST conversion to SNP based molecular markers and their use in saturation or comparative mapping has been an important recent area of research and several techniques for SNP analysis have been reported [12]. For example, in common bean three methods have been used for EST marker conversion based on SNP polymorphisms. In the first, cleaved amplified PCR fragment techniques (CAPS and dCAPS) were used to convert EST based polymorphisms into genetic markers [13]. A second attempt involved a high-throughput system named Luminex-100 which was used to confirm SNP calls in DNA from 10 common bean genotypes, finding 2.5% of SNPs were miscalled and 1% had no signal as compared with direct sequencing [11]. In an effort to simplify SNP analysis, Galeano et al. [14] used CEL I mismatch digestions to analyze and map SNP-based, EST-derived markers, finding that the method worked well with SNPs located in the middle of amplification fragments and that digestion products could be visualized on agarose gels.

Some of these techniques require specialized equipment or ingredients, which some molecular marker laboratories may not have. Therefore, a recent goal in our laboratory has been to identify a gel-based alternative that does not require restriction enzyme digestion. In this regard, we have found single strand conformational polymorphism (SSCP) analysis to be a good alternative. The SSCP technique is based on conformational differences of single stranded DNA fragments that can be detected as mobility shifts in non-denaturing polyacryilamide gel electrophoresis [15]. This technique is easy and inexpensive to implement as we show in this study and has been used to analyzing gene or EST derived SNP markers in various plant species such as wheat (*Triticum aestivum *L.)[16,17], barley (*Hordeum vulgare *L.)[18], grapevine (*Vitis vinifera *L.)[3], cassava (*Manihot esculenta *Crantz)[19], pearl millet [*Pennisetum glaucum *(L.) R.Br.][20] and *Pinus *species [21].

In this study, our objective was to develop and map SSCP markers on an integrated genetic map for common bean using EST or gene-based markers from various sources. The molecular mapping of genic SNPs and Indels through this technique also provided the basis to analyze synteny of homologous loci across the legumes. In relation to this, the genetic map information and the marker sequences were used for an analysis of macro-synteny between the genome of common bean and the genomes of *Glycine max*, *Lotus japonicus *(*Lotus*) and *Medicago truncatula *(*Medicago*).

## Results

### Parental survey

**Figure 1:**
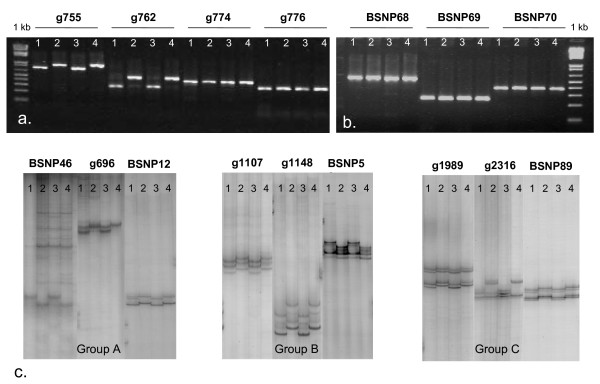
**Gel images for SNP and Indel based markers**. Evaluations of the PCR products representing SNP markers developed in this study: a) gene based amplicons on agarose showing length polymorphisms (g755 and g762) or monomorphisms (g774 and g776) when comparing genotypes DOR364, G19833, BAT93 and JaloEEP558 (lanes 1 to 4, respectively). b) EST based amplicons for BNSP68, BNSP69 and BNSP70 on an agarose gel with the same four genotypes (lanes 1 to 4). c) gene based amplicons evaluated in a silver-stained SSCP gel showing three examples of polymorphisms with the same genotypes (lanes 1 to 4) for groups A, B and C, corresponding to molecular weights of 50-200 bp, 200-600 and 600 -1000 bp, respectively.

After the standardization process, a total of 418 amplicons were successfully amplified on the four genotypes evaluated. Of these, 93 amplicons were derived from SNP containing EST sequences of Ramirez et al. [10] and corresponded to BSNP markers either newly developed here or from Galeano et al. [14], 300 amplicons were from the "g" series developed by NDSU and 25 amplicons were from the SNP containing fragments reported by Gaitán-Solís et al. [11]. Figure 1a shows examples of polymorphic and monomorphic PCR products evaluated on agarose gels for indel type size polymorphisms. In that figure, amplicons for g755 and g762 presented clear size polymorphisms between Andean genotypes (G19833 and Jalo EEP508) and Mesoamerican genotypes (DOR364 and BAT93), while the amplicons for g774 and g776 showed monomorphism in product size. The amplicons for BSNP68, BSNP69 and BSNP70 (Figure 1b) were also monomorphic in size (no indel) but as described in Additional File 1 were designed to cover the SNPs in contig 2624 from Ramirez et al. [10]. All of these amplicons are examples of the quality and specificity of the amplicon required to initiate the SSCP technique. In total, 11 amplicons showed size polymorphisms showing that indels are not frequent in the marker set used in this study.

SSCP polymorphism, meanwhile, was found in 106 out of the 418 amplicons tested in the parental survey for mapping parents used in this study. This included screening on MDE gels of all the amplicons that did not contain indel polymorphisms from the agarose gel screening. The overall SSCP polymorphism rate was similar for the parents of the populations DOR364 × G19833 (25.6%) and BAT93 × Jalo EEP558 (22%) with the former cross selected for genetic mapping. Figure 1c show examples of amplicons with clear conformational polymorphisms between the Andean and Mesoamerican genotypes using the SSCP technique. Among the polymorphic amplicons for the mapping population, 34 were for BSNP markers from Galeano et al. [14], 66 were for 'g' markers and 6 were for markers from Gaitán-Solis et al [11].

PCR product size was not found to affect SSCP detection in the BSNP markers and this method could be used for amplicons up to 1 kb in size. For example, Figure 1c shows the electrophoresis profile of parental genotypes divided into groups A, B and C consisting in light molecular weight (50-200 bp), medium molecular weight (200-600 bp) and high molecular weight (600 -1000 bp) products, respectively, proving the versatility of the MDE technique to detect SSCPs in amplicons with a wide range of molecular weights. Gel migration was lengthier for the larger molecular weight fragments than for the smaller molecular weight fragments with as little as four hours of run time needed for the first group and 16 hours needed for the third group. It was notable, that some amplicons for the BSNP markers presented molecular weights in agarose gel evaluation that were greater than expected, suggested the presence of intronic regions, even though primer design had been based on fragments of 500 bp or less. PCR products were obtained up to 1000 bp (eg. marker BSNP869) and the smallest amplicons were as small as 100 bp (marker BSNP5) and both extremes were equally amenable to SSCP evaluation.

### Molecular mapping and linkage analysis

**Figure 2:**
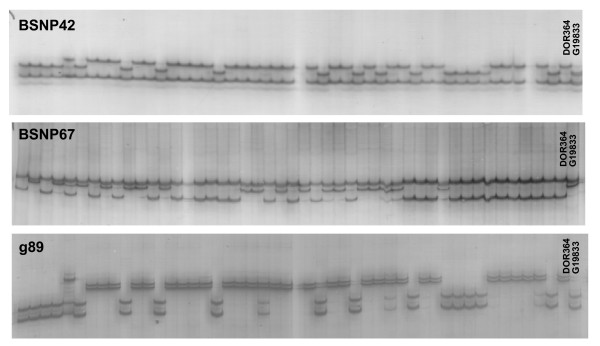
**Genetic mapping of SSCP based markers**. Segregation pattern of two BSNP markers and one "g" marker in the DOR364 × G19833 recombinant inbred line mapping population based on SSCP polymorphism and detection on silver stained gels.

After the parental screening, the polymorphic amplicons were evaluated as genetic markers for the entire DOR364 × G19833 RILs population. All 106 resulting loci evaluated with the SSCP technique were placed in the linkage map, along with a further 11 Indel based markers evaluated for segregation with agarose gels. Figure 2 shows examples of the SSCP based markers evaluated in the population having typical segregation pattern for parental alleles of 1:1 for a co-dominant marker evaluated in a recombinant inbred line population. In the case of the marker g89 a few heterozygous individuals were detected. Apart from the SSCP detection, one marker was mapped with CEL I heteroduplex digestion as reported by Galeano et al. [14] for a total of 118 markers evaluated in the population for the genetic mapping portion of this study. The 11 amplicons/markers evaluated with agarose gel evaluation were g128, g166, g755, g993, g1148, g1341, g1388, g2068, g2510, g2647, gCV542014 while the marker evaluated using CEL I heteroduplex digestion was BSNP78. Segregation of the new marker loci was analyzed along with the 165 SSR, STS and RFLP markers reported by Blair et al. [22,23], Caldas and Blair [24] and Beebe et al. [25] as well as five CEL I based gene markers reported by Galeano et al. [14].

**Figure 3:**
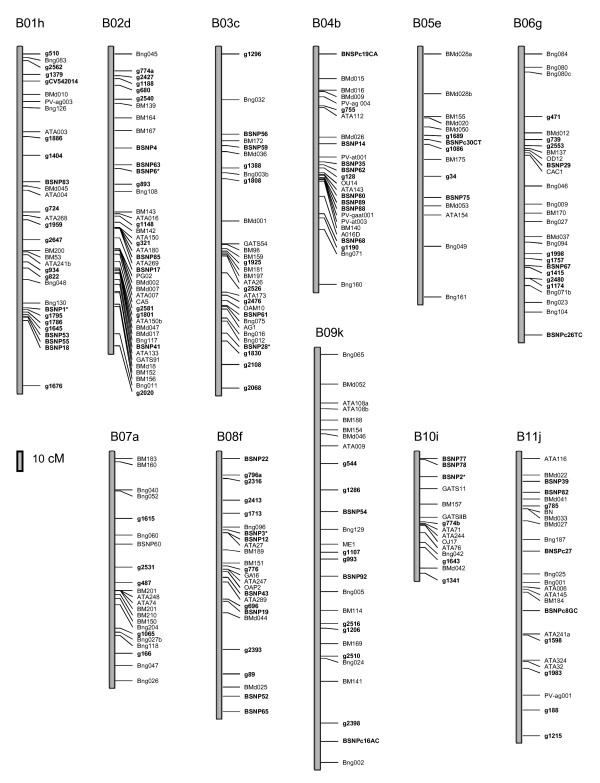
**Linkage analysis of SNP and Indel markers in the common bean genome**. Common bean linkage map for the DOR364 × G19833 recombinant inbred line mapping population. Chromosome designations are indicated above the linkage groups (b01 to b11). The 118 newly mapped markers are shown in bold. The markers mapped by Galeano et al. [14] have asterisks while other genetic marker loci positions are as reported in Blair et al. [22,23].

The final genetic map which is shown in Figure 3 and summarized in the Table 1 had 288 marker loci in total with a full map length of 1,900 cM. Linkage group sizes ranged from 75 cM (B10) to 268 cM (B09) with an average of 172 cM per linkage group. The number of marker loci per linkage group ranged from 15 on B05 to 43 on B02. The largest number of new marker loci (19) was placed on linkage group B01 but an average of 11 new loci were placed on each linkage group. The average distance between the EST-based marker loci and the SSR or RFLP marker loci was 13.5 cM and 10.6 cM, respectively and the average distance between all loci was 6.8 cM. In general, the marker loci were well distributed within the linkage groups; however, some markers clustered in certain regions of B01, B02 and B06. Segregation distortion was found for 19% of the new marker loci (based on Chi-square tests at P < 0.05) which is similar to values in Blair et al. (2003). In general the most highly distorted marker loci were found on B02 and presented preferential transmission of the G19833 allele. The same distortion was found in the middle of B01; while preferential transmission of the DOR364 alleles was found at the top of the B09.

**Table 1:** Summary of markers and genetic distances found for each common bean linkage group (LG).

LG	New markers	Total markers	Distance between new markers (cM)	Distance betwen all markers (cM)	Total distance (cM)
B01	19	33	10.86	6.45	206.29
B02	16	43	10.70	4.32	181.54
B03	12	30	17.29	7.15	207.44
B04	11	26	10.18	5.73	143.21
B05	5	15	9.66	10.79	151.00
B06	11	27	12.32	6.71	174.57
B07	6	22	17.72	6.94	145.67
B08	14	25	11.85	6.91	165.86
B09	11	27	18.23	10.29	267.65
B10	4	15	15.07	5.02	75.36
B11	9	25	20.86	7.57	181.69

Average	10.7	26.27	13.51	6.84	172.75

Total	118	288			1900.30

### Synteny analysis

**Figure 4:**
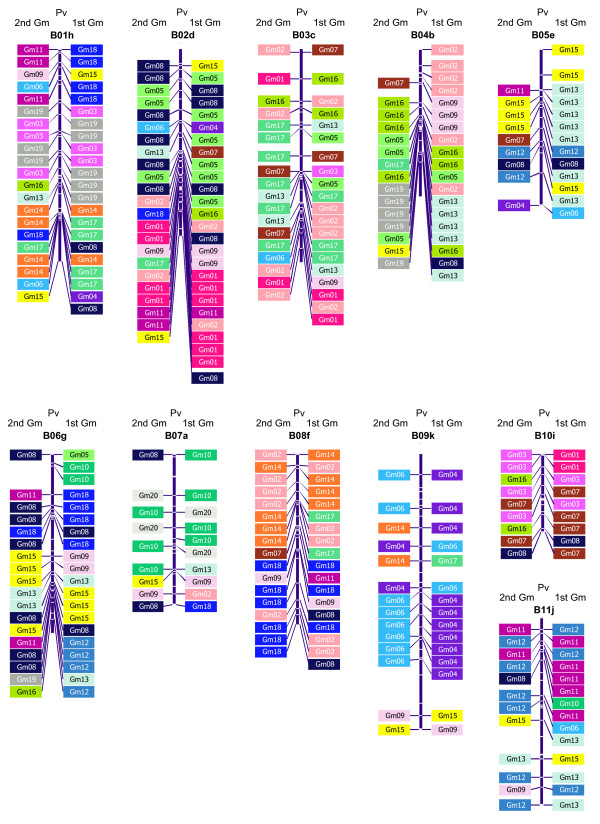
**Synteny relationships between common bean and soybean**. Associations between common bean and soybean linkage groups through sequence based markers. The colored boxes represent the homologies with chromosome segments from the soybean genome with each chromosome from soybean assigned a given color. The boxes to the right side of the linkage group are the first similarity matches, while to the left side are the second similarity matches.

A total of 218 markers were used for the synteny analysis based on sequences of EST contigs or singletons corresponding to the assembly of the new SSCP and Indel based markers or the sequences for SSRs from Blair et al. [22] and RFLPs from Murray et al. [26] with the EST collection of 83,448 sequences from GenBank. The resulting marker-based sequences were aligned against genomic sequences of soybean showing 186 significant homologies based on a first hit *E*-value average of 10^-13^. In addition, a second match was recorded for the blastn evaluation, because the soybean genome has had at least two rounds of polyploidization [27], and therefore presumably has at least two homologous copies of each common bean gene or EST represented in distinct positions of its genome. A total of 165 of the markers evaluated were found to have a significant second match with the soybean genome. The first hit markers from the homology search against the soybean genome were distributed across all 11 linkage groups of the common bean genetic map as shown in Figure 4 where the syntenic relationships for each marker are indicated with flanking boxes identifying the soybean chromosome where its homolog or homologs are found. On average 17 such conserved markers were present in each common bean linkage group with the most syntenic relationships discovered for B02 (26 markers) and the least for B10 (9 markers).

Comparative mapping and sequence positioning between common bean and soybean presented a mosaic pattern where it was possible to identify syntenic regions based on three or more markers matching from the same chromosome of soybean to the same region of a common bean linkage group. Through this analysis, most common bean linkage groups could be represented by several re-arranged soybean chromosomes as seen in Figure 4. For example, segments of linkage group B01 presented synteny with three pairs of soybean chromosomes (hereafter named Gm), namely the top segment of B01 with Gm18 in first hits and Gm11 in second hits, the middle segment with Gm19 and Gm3 and the bottom segment with Gm17 and Gm14. Linkage group B02 presented synteny with Gm5 and Gm8; and at the bottom with Gm1. Linkage group B03 was syntenic with Gm2 and Gm17 along most of its entire length; while B04 showed synteny with Gm2 at the top of the linkage group, with Gm16 in the middle and with Gm13 and Gm19 at the bottom. Linkage group B05 had synteny with Gm15 and Gm13 although second hits were variable; while B06 presented a syntenic block with Gm18 and Gm8 at the top of the linkage group, with Gm15 and Gm13 in the middle and with Gm12 at the bottom. B07 showed a syntenic block with Gm10 and Gm20 except at the bottom of the linkage group; while B08 showed synteny with Gm14 and Gm2 at the top, and with Gm18 at the bottom. Linkage group B10 showed a syntenic block with Gm3 and Gm7; while B11 was syntenic with Gm 11 at the top, and Gm13 in the bottom. Finally, B09 was mostly syntenic with a single pair of soybean chromosomes, Gm4 and Gm6, along its entire length except at the very end.

**Figure 5:**
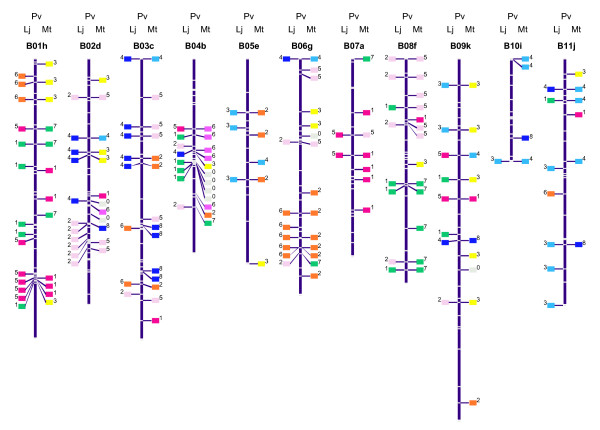
**Synteny relationships between common bean and *Lotus japonicus *and *Medicago truncatula***. Associations between common bean, *Lotus *and *Medicago *linkage groups through sequence based markers. The number in the right and left hand boxes indicate the chromosomes numbers of the *Medicago *and *Lotus *hits, respectively. Each chromosome from each legume was assigned a different color with colors chosen to cover a large spectrum for ease of visualization.

The synteny analysis with *M. truncatula *and *L. japonicus *resulted in totals of 109 and 78 homologous markers linked to the common bean genome, respectively. These represented averages of 10 and 7 anchor markers per linkage group in *P. vulgaris *for the two model legume genomes, respectively. As seen in Figure 5, linkage group B01 showed various syntenic blocks with *L. japonicus *(hereafter named Lj), or *M. truncatula *chromosomes (hereafter named Mt). Linkage group B02 showed syntenic blocks with Lj2 and Lj4, but no clear synteny with the *Medicago *chromosomes. Linkage group B03 showed segmental synteny with Lj4, Mt5 and Mt8. B04 showed syntenic blocks from Lj1 and Mt6, and interestingly with Mt0, a "false" chromosome where unanchored sequences from the *Medicago *genome project are temporarily housed. Linkage group B05 showed a syntenic block with Lj3; while B06 showed a syntenic block with Lj6 and Mt2. Linkage group B07 showed a syntenic block with Mt1; B08 with Mt5 and Mt7 and B11 with Lj3. Meanwhile, linkage groups B09 and B10 did not have a clear synteny with the other legume genomes. In summary, the synteny analysis of common bean with the genomes of *Medicago*, *Lotus *and soybean showed that 50%, 36% and 85% of the bean anchor markers had similarity with genes from these species, respectively.

## Discussion

### Value of the SSCP based markers for map saturation

This study creates a transcript map for common bean based on markers evaluated with SSCP gels which was found to be an easy to use marker system to screen for single base substitutions and SNP polymorphisms as well as small insertion-deletion events. In terms of marker development and testing, a total of 93 amplicons have now been developed as part of the BSNP series which we began the development of for the analysis of CEL I assays as described in Galeano et al. [14] and which are based on the 138 doubly-confirmed SNPs detected in Ramirez et al. [10]. In addition we tested a total of 325 amplicons for SSCP and Indel polymorphisms based on the gene-derived markers from Gaitan-Solís et al. [11] and the EST derived markers from NDSU [28]. Genetic mapping was shown to be successful with the SNP and Indel detection techniques used in this study. Out of the full set of 418 amplicons tested, a total of 118 could be located on the genetic map of common bean using the inter-genepool population DOR364 × G19833 creating a more saturated genetic map for common bean.

The new map presents important advantages since it is based on a combination of cDNA-based and non-gene markers, integrating the 118 new EST and gene-based marker loci into a map that already had 165 SSR, STS or RFLP markers for this population from Blair et al. [22,23], Caldas and Blair [24] and Beebe et al. [25]. The result is one of the most dense as well as most diverse molecular maps available for common bean to date with a total of 288 single copy markers mapped in this population and a full set of 451 markers for the population if we include AFLP, RGA, RAPD and SCAR markers also mapped in this population as described in those previous publications. The success rate for the amplicons tested in this study in terms of mapping with the SSCP detection techniques was between 47% and 22% depending on the source of the markers. For example, of the 72 SNP-EST contigs reported by Ramirez et al [10] we were able to map 34 in this study building on and improving over the initial mapping conducted in our earlier study (Galeano et al. [14]. Out of the 25 amplicons developed by Gaitan-Solis et al. [11] we were able to place six markers through the SSCP technique with the main advantage being that SSCP detection is technically easier than the multiplex method used by these authors. Finally, we were able to place 66 other gene based markers through SSCP detection and 11 through indel evaluation on the DOR364 × G19833 population based on marker development at NDSU for the g series of SNP markers. These latter markers have also been mapped in the BAT93 × Jalo EEP508 as reported in the Legume Information System [28] and are on equivalent linkage groups with highly correlated marker order between both genetic maps. Therefore, the present genetic map is a fully integrated, centralized map for common bean given its high marker saturation.

Several advantages of our SSCP technique as an option for SNP and Indel discovery and genetic analysis were noted. First, this technique did not require the special inputs used by other SNP detection techniques [20] except for the MDE component of SSCP gels and could be undertaken with standard equipment and PCR setups found in most labs along with the same infrastructure and procedures used for SSR electrophoresis [22]. Secondly, our SSCP evaluations were carried out with electrophoresis conducted at a room temperature of 18 to 20°C rather than refrigerated at 4°C showing that results are not sensitive to higher temperatures or to small temperature fluctuations. The MDE matrix has been reported previously as not being sensitive to temperature [20,29]; nevertheless, some SSCP studies have found temperature effects on fragment migration and gel resolution when using acrylamide gels [15,16]. The reason for this sensitivity could be explained because higher temperatures might destroy some semi-stable conformations [15]; however, the MDE gel appeared to produce a more stable behavior of DNA conformations.

Polymorphism discovery through SSCP technology offers a valuable tool for genetic which in the future can be complemented by targeted approaches that increase the chances of finding SNP polymorphisms for a given PCR product and given cross combination. While SNP frequency seems to be high in inter-genepool comparisons as we showed here for DOR364 and G19833, it appears to be lower in within genepool comparisons for example among two Andean genotypes [30]. One alternative is to design primer pairs surrounding intron sequences that tend to be more variable than exons as has been reported by Choi et al. [31] and Bertioli et al. [32]. Another option is to employ next generation sequencing prior to SSCP marker design so as to rapidly many SNPs between individuals [33]. While common bean has not been the subject of next-generation sequencing yet, the range of EST sequences found for the crop is a valuable source for further SNP discovery. In addition since common bean is known to be very diverse based on SSR marker analysis of the two major gene pools [34-36] analysis of sequence information from multiple genotypes is likely to generate new sources of SNPs useful for marker conversion and SSCP analysis.

### Utility of the transcript map for synteny analysis

Apart from the development of SSCP markers, our mapping of EST-based sequences allowed us to embark on comparative mapping within the legumes and across the Papilionoideae subfamily which contains the most important group of crop legumes within two specific clades that can benefit from cross-species analysis [37,38]. The galegoid clade, including the tribes Viceae, Trifolieae, Cicereae and Loteae and the genera *Vicia*, *Medicago*, *Cicer *and *Lotus *is made up of temperate species; while the phaseoloid clade, which is synonymous with the tribe Phaseoleae, includes the genera *Phaseolus*, *Vigna *and *Glycine *and crops such as common bean, lima bean (*P. lunatus)*, tepary bean (*P. acutifolius*), cowpea (*Vigna unguiculata*) and soybean [39]. In this study, we took advantage of the nearly full genome sequences now available for soybean, *Medicago truncatula *and *Lotus japonicus *[40,41] to conduct macrosynteny analysis with common bean based on homology searches for the sequenced markers on our genetic map. Similar studies have been conducted using orthologous markers with or without sequence information across various crop and model legumes by Bertioli et al [32]. Cannon et al. [42], Choi et al. [31,37], Hisano et al. [43], Hougaard et al. [13] and Tsubokura et al. [44].

The synteny results from our study comparing common bean linkage groups with soybean chromosomes showed large macrosyntenic segments between parts of 'homeologous' chromosome pairs from the ancestrally polyploidy genome of soybean syntenic and different parts of the diploid common bean genome. This allowed us to align most of the 20 soybean chromosomes to the 11 individual linkage groups of common bean in paired segments as shown in Additional file 2 (dotblot). The syntenic blocks found between common bean and soybean Gm10-B07, Gm16-B03, Gm5-B02, Gm19/Gm15/Gm12-B06, Gm18-B01, Gm3-B10, Gm12/Gm11-B11 and Gm6-b09 were consistent with the RFLP based comparisons of the linkage maps of soybean, common bean and *Vigna radiata *[45]. In addition, the specific synteny of Gm11/Gm12 with B11 was corroborated by Lee et al [46] comparing the genome organization of these same three legumes around the genes *Pa*1 and *Pa*2 which encode pubescence in soybean.

Our results agree with previous studies suggesting that the soybean genome has undergone two or more large scale duplications and is probably an ancient polyploid [47]. Based on the common bean model, we identify some of the duplicate chromosome regions in soybean. For example, linkage group B07 showed synteny with regions of both Gm10 and Gm20 (corresponding to soybean linkage groups O and I). Duplication of this chromosome from soybean was found when mapping BACs containing paralogous ω-6 fatty acid desaturase (FAD2) genes [48]. I n addition, our synteny results for linkage group B09 infer a duplication of Gm4 and Gm6 (C1 and C2) as was also found by Schlueter et al. [49] when sequencing homeologuos BACs anchored by mapped duplicate *N-*hydroxycinnamoyl benzoyltransferase (HCBT) genes. Other soybean chromosome duplications we detected included Gm13-Gm 19 (N and L), Gm8-Gm5 (A2 and A1), Gm13-Gm15 (F and E), Gm18-Gm8 (G and A2), Gm2-14 (D1b and B2), Gm4-Gm6, and Gm11-Gm12 (B1 and H). These results are consistent with the duplication analysis carried out by Shoemaker et al. [47] using RFLP markers and the synteny results of Hisano et al [43], and Tsubokura et al. [44]. Our study, therefore, confirms results of Shoemaker et al. [50], showing that compared to common bean, the soybean genome is the result of a duplicated ancestral genome that was re-arranged to produce 20 non-homologous chromosomes with many homeologous regions among chromosome pairs. So far, macro-synteny studies among other legumes have revealed that the genome structure is relatively stable within the subfamily Papilionoideae albeit with many of these segmental rearrangements [31,42].

The synteny analysis with *Medicago *and *Lotus *compared to the linkage groups in common bean identified many more rearrangements. Despite this, chromosomes Lj4 and Lj2 were found to be related to linkage group B02, Lj4 and Mt8 to B03, Mt2 and Lj6 to B06, Mt1 to B07, Mt7 and Mt5 to B08, Mt5 to B01 and B02, Mt3 to B09, Lj3 to B10 and Mt4 to B11. Similar results are reported by Hougaard et al. [13] who analyzed 99 and 75 shared loci, respectively, between *Lotus *or *Medicago *and common bean and by Choi et al. [37] who compared various galegoid and phaseoloid genomes using cross species genetic markers. Results comparing soybean with *Lotus *by Hisano et al. [43] and Tsubokura et al. [44] found a limited degree of macrosynteny between these species, perhaps because of the complex structure of the soybean genome compared to the simpler structure of the *Lotus *genome. Interestingly, in both our study and the one from Hougaard et al. [13], it was almost impossible to find synteny for linkage groups B05 and B10 due to few anchor markers in these regions suggesting that these linkage groups have lower number of conserved or transcribed sequences.

The correspondence of our results with the synteny blocks (SB) of Cannon et al. [42] appear to be most robust on B01 and B07 for sequences from SB1 and SB2 (Lj5/Mt1), on B06 for SB3 (Lj6/Mt2), and on B08 and B02 for SB9 and SB10 (Lj2/Mt5), respectively. Synteny blocks were also evident in the studies of Young et al. [41] and Hougaard et al. [13]. Meanwhile, Bertioli et al. [32] identified differences in syntenic blocks between peanut (*Arachis *spp.) and *Medicago *or *Lotus*, finding that retrotransposon-rich regions are distributed in alternating blocks across these legume genomes and that these are interspersed between syntenic blocks and correspond to the variable regions which do not show synteny.

Similar results in comparative mapping using ESTs and gene based markers have allowed the identification of homologous linkage groups in studies of related *Pinus *species [51], or across *M. truncatula *and *M. sativa *[31]. Comparative mapping across distantly related species is also possible with conserved markers used to compare the sugar beet transcript map with the *Arabidopsis *genome [52] or sequence comparisons of *Arabidopsis *and rice [53] with a few syntenic blocks always found, suggesting limited co-linearity between distant dicotyledonous or angiosperm genomes. The long time of divergence (50 Mya) between galegoid and phaseoloid clades [54] would explain the less frequent homologies between common bean markers and *Lotus *or *Medicago *genomes compared to hits with the soybean genome. Our results confirm the reduced level of conservation between galegoid (*Medicago*, *Lotus*) and phaseoloid (common bean, soybean) legumes as reported Choi et al. [55] concluding that synteny is high among closely related species, and that the degree of synteny declines with increasing phylogenetic distance.

## Conclusions

In summary, the SSCP technique in common bean was found to be a useful alternative marker system for the genetic analysis of EST-based amplicons and SNP or Indel based polymorphisms. We reported the versatility of this technique given its capacity to analyze a wide range of PCR fragment sizes using simple equipment and standard conditions. The SSCP technique was especially useful for saturating the common bean map with gene derived marker loci, and the resulting transcript map was then used for macrosynteny analysis with soybean, *Lotus *and *Medicago *genomes. We expect that the EST and gene based map will be useful for positional cloning and for dissection of quantitative traits, and the identification of the genes underlying these. In this regard the enhanced map for DOR364 × G19833 may provide us with the tools for map-based cloning of QTL for low phosphorus tolerance, high nutritional quality and other high priority traits discovered by our laboratory for this population [23,25,56,57]. In addition, the markers will be useful as tools for marker assisted selection in common bean and for the further analysis of phylogenetic relationships and conserved regions between the genome of this crop and those of model legumes [58]. Furthermore, these results offer a valuable framework for utilizing sequence information from soybean and model legumes for further marker development and gene characterization in common bean.

## Methods

### Plant materials

A parental survey was conducted with the Andean genotypes (landraces) G19833 and Jalo EEP558 and Mesoamerican genotypes (varieties) DOR364, BAT93. The Mesoamerican genotypes DOR364 and BAT93 are both improved lines from the International Center for Tropical Agriculture (CIAT) while the Andean genotypes G19833 and Jalo EEP558 are a landrace from Peru and a released variety from Brazil, respectively. The molecular mapping was then conducted with the 87 recombinant inbred line (RIL) progeny from the cross DOR364 × G19833 whose development and origins are described in Blair et al. [22,35], or Beebe et al. [25] and which is in the F_9:11 _generation. The DNAs of the parents and the RILs were extracted using a CTAB extraction procedure described in Afanador and Hadley [59].

### Marker sources

A total of 418 amplicons were tested for SSCP or Indel polymorphisms, including 56 from primer pairs newly designed for this study (Additional file 1) based on SNP polymorphisms between Negro Jamapa and G19833 leaf ESTs libraries reported by Ramirez et al. [10], 37 amplicons developed by our laboratory for EST derived SNPs as described previously [14], 25 SNP based markers from Gaitán-Solís et al. [11] and 300 "g" markers from North Dakota State University (NDSU) that have been described in the Legume Information System [28]. The development of the new bean SNP markers (BSNP) was based on conditions for marker design as given in Galeano et al. [14] with the design of BSNP markers targeting amplification products with an average size of 200 bp that were all smaller than 500 bp based on original EST contigs from Ramirez et al. [10].

### PCR amplification and agarose gel evaluation

All PCR reactions were carried out in 15 μl reaction volumes containing 25 ng of genomic DNA, 0.2 μM each of forward and reverse primers, 20 mM of total dNTPs, 1× PCR buffer [10 mM of Tris-HCl (pH 7.2), 50 mM of KCl], 2.5 mM MgCl_2 _and 1 unit of *Taq *polymerase. The amplification protocol consisted of 34 cycles of 30 s at 94°C, 40 s at 50 to 60°C (depending on the annealing temperature of each primer as given in Additional file 1), and 2 minutes at 72°C, followed by 5 minutes extension at 72°C. The PCR products combined 3:1 with loading buffer (30% glycerol and 0.25% bromophenol blue) were then run in 0.5× TBE buffer on 2% agarose gels in HORIZON 20:25 gel electrophoresis system units (Gibco BRL Life Technology Inc., Gaithersburg, MD). The gels were stained with ethidium bromide and visualized on an ultraviolet trans-illuminator with documentation by a Gel-Doc 2000 photosystem (Bio-Rad Laboratories, Richmond, CA) to confirm amplicon quality, molecular weights and any observed length polymorphisms.

### SSCP marker assay

The PCR products were denatured and separated on SSCP gels containing a mutation detection enhancement (MDE) solution as described by Castelblanco and Fregene [19]. The gel was made up of 6 mL TBE buffer (5×), 39 mL deionized water and 15 mL MDE gel solution (Cambrex Biosciences Rockland, Maryland) which was polymerized by the addition of 0.3 mL ammonium persulphate (10%) and 30 μL tetramethylenediamine (TEMED). The PCR product was mixed with 5 μL of loading dye, denatured at 95°C for 5 min, cooled on ice. The electrophoresis run times were for 4, 10 and 16 h depending on the molecular weight of the fragment being analyzed. Constant power of 8 W was used to run the gels at room temperature in Sequi-Gen GT 38 × 30 cm electrophoresis units (Biorad, Hercules, Calif., USA). Silver staining was performed as described by Blair et al. [22].

### Linkage analysis

Segregation data was used to place the new markers on the established genetic map for the DOR364 × G19833 population described in Blair et al. [23]. Linkage analysis was conducted with the Kosambi mapping function using the software application Mapmaker 2.0 for Windows [60]. The markers were placed to the most-likely interval with the 'try' command and a minimum LOD of 3.0. Marker order was then determined by multipoint analysis and was confirmed with the 'compare' command using a minimum LOD of 4.0. To generate a more reliable map for the DOR364 × G19833 population, sequence characterized amplified region (SCAR) and random amplified polymorphic DNA (RAPD) markers were removed from the matrix and only the simple sequence repeat (SSR), sequence tagged site (STS) and restriction fragment length polymorphism (RFLP) markers were used as frameworks for the new EST and gene based SSCP markers. The linkage group were named as reported in Blair et al. [22] whereby the naming system from the reference RFLP maps published by Freyre et al. [61] and Vallejos et al. [62] are combined.

### Synteny analysis

Sequences of the SSR, RFLP and new SNP markers were downloaded in FASTA format from NCBI. The sequences of the BSNP and g markers were assembled with all available ESTs for common bean (83,448) as of July 2009 using EGassembler [63] to use longer sequences from the resulting contigs in homology searches. SSR sequences were masked for simple repeats and low complexity regions with RepeatMasker, an on-line tool from Institute for Systems Biology [64]. The sequences were aligned with an *E*-value threshold of 1 × 10^-10 ^against the chromosome-based assembly of soybean, Glyma1.0, developed by the Department of Energy's Joint Genome Institute and the Center for Integrative Genomics [65] using local blastn that had been downloaded from NCBI. The same process was used to align markers to the *Medicago *genome release version 2.0 [66]. Meanwhile, for *Lotus *versus common bean comparisons markers were aligned against the genome structure reported by Sato et al. [40]. Graphics were drawn with an in-house software created with Visual Basic Script programming language in a Microsoft Excel™ environment based on the following information: marker name, linkage group in common bean and cumulative distance (in cM) on that linkage group, GenBank accession number or contig name, chromosome in the model legumes and position (in bp) on that chromosome. Dotplots of marker positions on the genomes of common bean versus the three other legumes were obtained with drawing formulas as described by Bertioli et al. [32].

## Authors' contributions

CHG participated in the conception of the study, carried out the genotyping and map construction and drafted the paper. ACF did all programming and design of computational experiments. MG participated in marker development from SNP-containing contigs. MWB conceived of and coordinated the study, interpreted results and co-wrote the paper. All authors read and approved the final manuscript.

## Supplementary Material

Additional file 1 EST-based amplicons designed for SNP containing contigs reported by Ramirez et al. [10], their primer sequences and melting (Tm) and annealing (Ta) temperatures and whether polymorphism (P) by SSCP was detected in the DOR364 × G19833 mapping population as well as the best hit to the Uniref protein database. Click here for file

Additional file 2 Dotplot of homologies between common bean markers from linkage groups B1 through B11 and sequences from soybean chromosomes Gm01 through Gm20. Click here for file
