# Supplementary material for: Single strand conformation polymorphism based SNP and Indel markers for genetic mapping and synteny analysis of common bean (Phaseolus vulgaris L.)
Source: BMC Genomics. 2009 Dec 23;10:629. doi: 10.1186/1471-2164-10-629 (PMC2806352; doi:10.1186/1471-2164-10-629)
Supplement: Additional file 1 — EST-based amplicons designed for SNP containing contigs reported by Ramirez et al. [10], their primer sequences and melting (Tm) and annealing (Ta) temperatures and whether polymorphism (P) by SSCP was detected in the DOR364 × G19833 mapping population as well as the best hit to the Uniref protein database. [file 1471-2164-10-629-S1.DOC]

**Additional File 1. Title:** Primer sequences and blast hits of newly designed BSNP markers.

**Description:** EST-based amplicons designed for single nucleotide polymorphism containing contigs from common bean reported by Ramirez et al. [10], their primer sequences and melting (Tm) and annealing (Ta) temperatures and whether polymorphism (P) by SSCP was detected in the DOR364 x G19833 mapping population as well as the best hit to the Uniref protein database.

| **Name** | **Contig ID** | **Forward primer** | **Tm (°C)** | **Reverse primer** | **Tm (°C)** | **Ta (°C)** | **uniref 100** | **P** |
| --- | --- | --- | --- | --- | --- | --- | --- | --- |
| BSNP38 | 2071 | CAAATCCCAAACGGAACAAACC | 55.2 | TAGTGATTTTCTCCGGCGCTTC | 57.4 | 52 | 50S ribosomal protein L12, chloroplast precursor [*Nicotiana tabacum*] | - |
| BSNP39 | 2071 | GCGCCGGAGAAAATCACTAAGC | 58.8 | CAGGCTCGTCAACGCTCTTACA | 59.2 | 65 | ribosomal protein L12, chloroplast precursor [*Nicotiana tabacum*] | + |
| BSNP40 | 2079 | GATCTGAACACTCTTGGAAGTTAAA | 53 | CGAGACCACCTTCATCCATA | 53.7 | 60 | ATP citrate lyase b-subunit [*Lupinus albus*] | - |
| BSNP41 | 2079 | CACTTTTCTTGGATGGCTTGAA | 53.8 | GACTGGTATATGCAAAGAGGCAAT | 55.2 | 53 | ATP citrate lyase b-subunit [*Lupinus albus*] | + |
| BSNP42 | 2401 | CATGGAAACTGGCATAGCAT | 53.2 | CCTGAAGCCAAAAGATGGAG | 53.6 | 55 | Sedoheptulose-1,7-bisphosphatase, chloroplast precursor [*Arabidopsis thaliana*] | - |
| BSNP43 | 2401 | CATCTCTCCATCTTTTGGCTTC | 53.6 | AGTGAAGTACCCTTTGCCTTTG | 55.3 | 53 | Sedoheptulose-1,7-bisphosphatase, chloroplast precursor [*Arabidopsis thaliana*] | + |
| BSNP44 | 2401 | CCTCACAAAAGCAACACCAGAT | 55.8 | CTCGTCCCCAAAAGAATTAACG | 53.9 | 53 | Sedoheptulose-1,7-bisphosphatase, chloroplast precursor [*Arabidopsis thaliana*] | - |
| BSNP45 | 2455 | CCCTCTCTCTCATCAACCAGTA | 55.3 | GTGGCTCACTTCTCTTACATACAC | 55.1 | 52 | Putative 21kD protein precursor [*Medicago sativa*] | - |
| BSNP46 | 2468 | CGTTCTCTTCTCTGCTCTCACG | 57.1 | GGGGGTAATGAGGGTGAACAT | 56.8 | 60 | Putative RNA binding protein [*Nicotiana tabacum*] | - |
| BSNP47 | 2517 | CCAAAAGGCACCGTCAGATATT | 55.4 | GTTCACTCCATCACACCCAATC | 55.9 | 64 | Monooxygenase 2 [*Arabidopsis thaliana*] | - |
| BSNP48 | 2517 | GGCCTCTTTTACTGGGAGACAT | 56.6 | GGAATAGCACCAGCTCGAATAC | 55.4 | 55 | Monooxygenase 2 [*Arabidopsis thaliana*] | - |
| BSNP49 | 2535 | GCTATGAAGAAATGGTGGATGC | 54.2 | CTTTATCCGCTTCACACTCACA | 55 | 55 | 14-3-3-like protein C [*Glycine max*] | - |
| BSNP50 | 2540 | GAAAGGGTGTGAGAGGTTTTGT | 55.3 | GTGGCTCCTTGTAGTCCTTGTC | 57.4 | 58 | Putative aquaporin PIP1-3 [*Vitis berlandieri x Vitis rupestris*] | - |
| BSNP51 | 2540 | GGCTTTGAGGGTAATGGTAGGT | 56.7 | ATGGGAAGTGGAGCCAAAATAG | 55.1 | 60 | Putative aquaporin PIP1-3 [*Vitis berlandieri x Vitis rupestris*] | - |
| BSNP52 | 2543 | CCACACAGTCCCCATCTATGA | 56.1 | GCAACTCGTAGCTCTTCTCCAC | 57.5 | 55 | Actin 11 [*Arabidopsis thaliana*] | + |
| BSNP53 | 2546 | CTTCTCACTCGCTGTCTCAATC | 55.2 | TTGTGTTGGCTTCCTTTGC | 54.1 | 55 | L-asparaginase [*Glycine max*] | + |
| BSNP54 | 2546 | GGGGACTGATGAACAAAATGA | 52.9 | TTGAACCCACAAGCCACTTC | 55.7 | 60 | L-asparaginase [*Glycine max*] | + |
| BSNP55 | 2546 | GAAGGAAAAGCTGGCCTCAT | 55.4 | GCACCACACACCAATTCAAA | 54.2 | 60 | L-asparaginase [*Glycine max*] | + |
| BSNP56 | 2558 | CTGCTTTCACAAATGGCTGCT | 56.6 | TCTCCCTCGGTGCTGAAGAC | 58.8 | 60 | Ferredoxin--NADP reductase, leaf isozyme, chloroplast precursor [*Pisum sativum*] | + |
| BSNP57 | 2558 | CATTGGTGACTTTGGTGATTCC | 54.3 | GGGTATTTCTCCTGCATCTTTTC | 53.8 | 60 | Ferredoxin--NADP reductase, leaf isozyme, chloroplast precursor [*Pisum sativum*] | - |
| BSNP58 | 2558 | GCAGAGAACAAACAAATGAGCA | 54.2 | GCAGGAGGGGTGATAGACTTG | 57.1 | 55 | Ferredoxin--NADP reductase, leaf isozyme, chloroplast precursor [*Pisum sativum*] | - |
| BSNP59 | 2566 | CGACGAAATCCAGAGCAAGAC | 55.9 | GCAGGCAAAGCAACAGCAT | 57.2 | 60 | Oxygen-evolving enhancer protein 1, chloroplast precursor [*Pisum sativum*] | + |
| BSNP60 | 2571 | CTTCTCAAACCTTCTGCTGTTC | 53.9 | TCACATAGAACTCCTCCACCTG | 55.8 | 55 | PSI-D1 precursor [*Nicotiana sylvestris*] | + |
| BSNP61 | 2579 | CATGGTTCCAGTTCGTGTTCA | 55.4 | CCAGTTCACTCGCTACAATGC | 56.2 | 60 | S-adenosylmethionine synthetase [*Phaseolus lunatus*] | + |
| BSNP62 | 2591 | GGTTATCTTGATGGGTGCAG | 53.4 | GATAGTGCAACATTCCTTCCA | 52.7 | 60 | LHCII type I chlorophyll a/b-binding protein [*Phaseolus aureus*] | + |
| BSNP63 | 2593 | GAGTTGGCCTTTGGTCCTTG | 56.4 | TGCATTGGCTGAAGAGTTGG | 55.8 | 55 | Photosystem I-N subunit [*Phaseolus vulgaris*] | + |
| BSNP64 | 2593 | GAAAAGAGCAAGACCAACAAGG | 54.4 | CATGTAAACGAAAGAGGCGAGT | 55.2 | 55 | Photosystem I-N subunit [*Phaseolus vulgaris*] | - |
| BSNP65 | 2599 | GCGAAGACTGTGGAGGTTGAG | 57.8 | GCCAGGTCAGCAAGAGCAATA | 57.6 | 65 | 14-3-3-like protein A [*Glycine max*] | + |
| BSNP66 | 2606 | TCTCAAAGCCACCGCCATC | 58 | GGTCCCACCAACACAAGCAT | 58.2 | 60 | Eukaryotic initiation factor 4A-9 [*Nicotiana tabacum*] | - |
| BSNP67 | 2623 | CTCACACTTCACATCCACTCACACA | 58.7 | CTTGCTCTCAACCCATCCCATAAG | 57.5 | 60 | Chlorophyll a/b-binding protein CP24 [*Phaseolus aureus*] | + |
| BSNP68 | 2624 | GGCTCTCTCTTCACCGTCAC | 57.5 | CAATGCGATAACCCTCCACT | 54.8 | 65 | LHCII type I chlorophyll a/b-binding protein [*Phaseolus aureus*] | + |
| BSNP69 | 2624 | GCTTTTGCTGAGTTGAAGGTTA | 53.7 | GAGCCGATAAAATTCACAATTC | 50.2 | 55 | LHCII type I chlorophyll a/b-binding protein [*Phaseolus aureus*] | - |
| BSNP70 | 2624 | GGTTATATTGATGGGTGCAGT | 52.5 | AGAGCCGATAAAATTCACAA | 49.6 | 55 | LHCII type I chlorophyll a/b-binding protein [*Phaseolus aureus*] | - |
| BSNP71 | 2627 | GGTGCCTCCTTCGTCACAG | 58.2 | GCTGGTCACATGGCCTAATG | 56.2 | 57 | Triose phosphate/phosphate translocator, chloroplast precursor [*Pisum sativum*] | - |
| BSNP72 | 2627 | CTTTGTGGCTGTCTCTGG | 53.2 | CAGTACGTTCCCAACTGC | 53.4 | 60 | Triose phosphate/phosphate translocator, chloroplast precursor [*Pisum sativum*] | - |
| BSNP73 | 2637 | GCTTTGATCCTCTCGGTCTTTC | 55.6 | TCAACTCCTTGAGCTTCTCTGG | 56.4 | 60 | Chlorophyll a-b binding protein 7, chloroplast precursor [*Lycopersicon esculentum*] | - |
| BSNP74 | 2641 | GGTGACTACGGATGGGACACAG | 59.2 | GGTCAAAGGAACCTCCAGGGTAG | 59 | 55 | LHCII type II chlorophyll a/b-binding protein [*Phaseolus aureus*] | - |
| BSNP75 | 2652 | GAAGTCATGGCAGCAACATTGA | 56.1 | TGAAGGGGTTTGAGCTGAGAAG | 57 | 65 | LHCII type III chlorophyll a/b binding protein [*Phaseolus aureus*] | + |
| BSNP76 | 2652 | GTAGGAGAGGGCAACAACCTTTA | 56.5 | TCTCATTGGAAAAAGAGGCTTAGTC | 54.9 | 60 | LHCII type III chlorophyll a/b binding protein [*Phaseolus aureus*] | - |
| BSNP77 | 2661 | GGAGCCTTATCGGGGACTA | 55.7 | ACCTCTTTTCTGGGTCAAGC | 55 | 60 | Chlorophyll a/b binding protein CP29 [*Phaseolus aureus*] | + |
| BSNP78* | 2661 | CAGAGATCAAGCACGCACGTC | 58.5 | AACTAATCCCCTGCCCCTTACC | 58.7 | 60 | Chlorophyll a/b binding protein CP29 [*Phaseolus aureus*] | + |
| BSNP79 | 2663 | CACACTCATCACCAATTCTCACA | 55.1 | CAAGGTAAGGGGGTCTAGGTTG | 56.8 | 60 | Photosystem I light-harvesting chlorophyll a/b-binding protein [*Nicotiana tabacum*] | - |
| BSNP80 | 2663 | AGAGGCATTGGGGTTAGGAAAC | 57.2 | CAGCCAAGTGAGTAGCCAAGTTC | 58 | 60 | Photosystem I light-harvesting chlorophyll a/b-binding protein [*Nicotiana tabacum*] | + |
| BSNP81 | 2664 | GAACCATCAAGCACCCTTCC | 56.2 | GCAAACACACCCTCACATCG | 56.7 | 60 | Cytosolic ascorbate peroxidase [*Vigna unguiculata*] | - |
| BSNP82 | 2668 | TGCTGTAGGAGATAAGATTGTGC | 54.6 | GGTGGATTGGTAGAGAGTTTCC | 54.9 | 60 | Plastidic aldolase [*Trifolium pratense*] | + |
| BSNP83 | 2670 | CCGCCGTTGATTGAAGATG | 54.2 | AGTGGAGGAGGTCACGAAGG | 58.6 | 60 | Nodulin-26 [*Glycine max*] | + |
| BSNP84 | 2670 | GCTTTGGTGTTGGAGATCGTG | 56.5 | GTGGGTGTGGCTGATGAAGAC | 58.2 | 60 | Nodulin-26 [*Glycine max*] | - |
| BSNP85 | 2672 | CAGGTGATTATGGTTGGGACA | 54.7 | CTGGGTAGATTGGGTCAGTCA | 56.1 | 64 | Photosystem II type I chlorophyll a/b-binding protein precursor [*Glycine max*] | + |
| BSNP86 | 2673 | CTGCTTCACTTCTGCTCCACA | 57.4 | TGAGAGGCTTGTTTCACACCAC | 57.5 | 60 | Glyceraldehyde-3-phosphate dehydrogenase A, chloroplast precursor [*Pisum sativum*] | - |
| BSNP87 | 2681 | CCGTGATGGTCGTATGGAGAAG | 57.2 | CCAAGAGCAGCCTCATTCAAGT | 57.5 | 60 | Ribulose bisphosphate carboxylase/oxygenase activase, chloroplast precursor [*Phaseolus vulgaris*] | - |
| BSNP88 | 2682 | CTCCTTTCACTGGGCTGAAGTC | 57.6 | CAAAGTGGGGTGGCTAGAACTG | 57.9 | 60 | Ribulose bisphosphate carboxylase precursor [*Phaseolus vulgaris*] | + |
| BSNP89 | 2685 | AGGAAATGGCTTCTTCAATG | 50.7 | GGTGACTTGTTGTGTTCACG | 54.3 | 55 | Ribulose bisphosphate carboxylase precursor [*Phaseolus vulgaris*] | + |
| BSNP90 | 2685 | GTACCGTGAACACAACAAGTC | 53.9 | GAAGTCCGAAAACAGAAACC | 51.6 | 55 | Ribulose bisphosphate carboxylase precursor [*Phaseolus vulgaris*] | - |
| BSNP91 | 2513 | GGTCCGGTTGAGAACTTAGC | 55.5 | GAGTTGGAACCCTCTCAGC | 55.3 | - | Type I (26 kD) CP29 polypeptide [*Lycopersicon esculentum*] | - |
| BSNP92 | 2583 | GGCTCAAACCATGTTCCTCA | 55.3 | GCAACACGACCCACAAAGAG | 56.7 | 55 | Photosystem II 22 kDa protein, chloroplast precursor [*Spinacia oleracea*] | + |
| BSNP93 | 2583 | GCTCTTTGTGGGTCGTGTTG | 56.7 | CTCTGAACCCTTTGCCTGAAG | 55.8 | 60 | Photosystem II 22 kDa protein, chloroplast precursor [*Spinacia oleracea*] | - |

* this marker was evaluated by CEL I technique as reported by Galeano et al. [14].
