# Supplementary material for: Single strand conformation polymorphism based SNP and Indel markers for genetic mapping and synteny analysis of common bean (Phaseolus vulgaris L.)
Source: BMC Genomics. 2009 Dec 23;10:629. doi: 10.1186/1471-2164-10-629 (PMC2806352; doi:10.1186/1471-2164-10-629)
Supplement: Additional file 2 — Dotplot of homologies between common bean markers from linkage groups B1 through B11 and sequences from soybean chromosomes Gm01 through Gm20. [file 1471-2164-10-629-S2.PPT]

## Slide 1
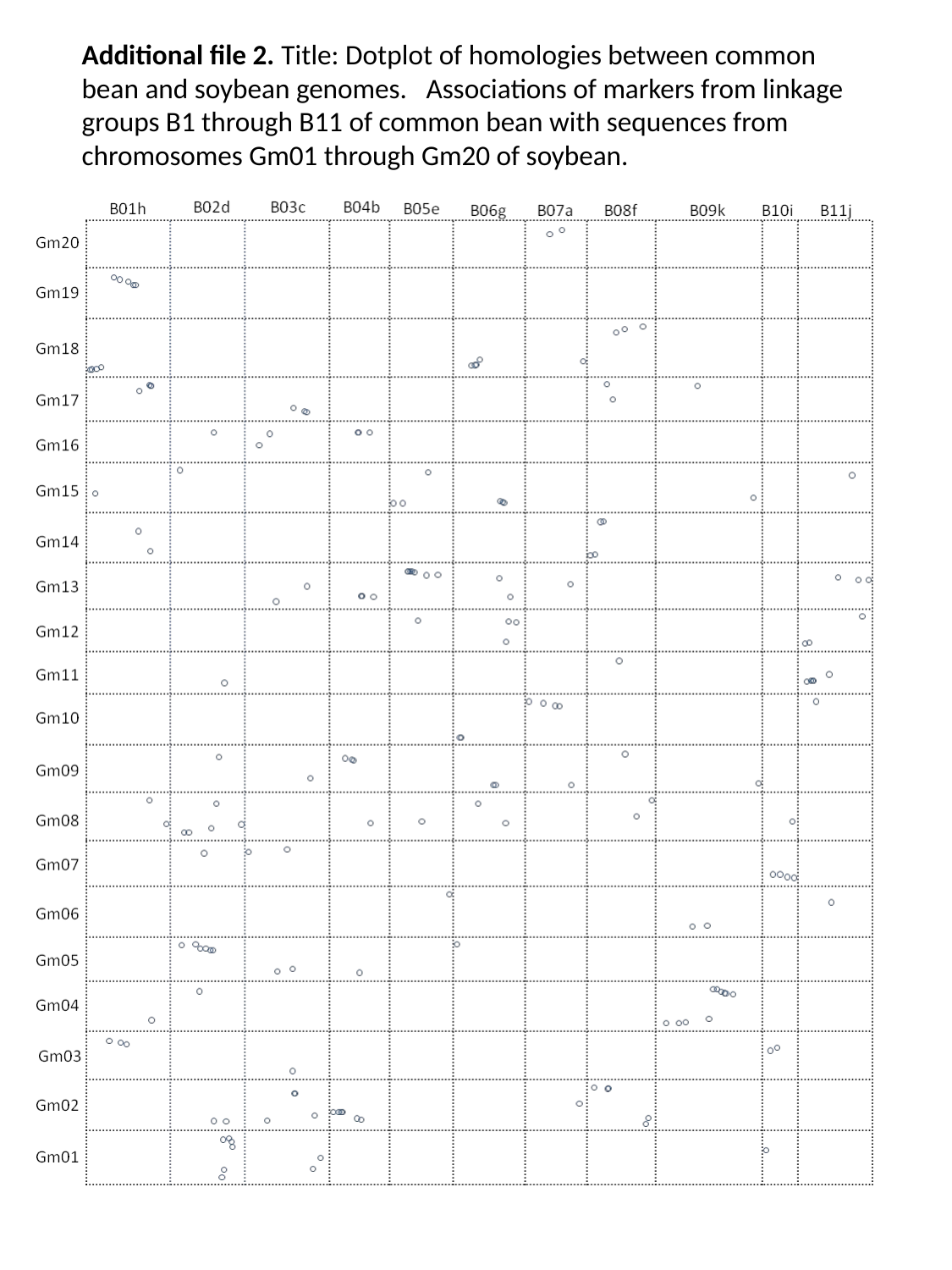

Additional file 2. Title: Dotplot of homologies between common bean and soybean genomes. Associations of markers from linkage groups B1 through B11 of common bean with sequences from chromosomes Gm01 through Gm20 of soybean.
